# Brace technology thematic series: the dynamic derotation brace

**DOI:** 10.1186/1748-7161-5-20

**Published:** 2010-09-21

**Authors:** Theodoros B Grivas, Achilles Bountis, Irene Vrasami, Nikolaos V Bardakos

**Affiliations:** 1 Department of Trauma and Orthopaedics, "Tzanio" General Hospital - NHS, Tzani & Afendouli str, 18536, Piraeus, Greece; 2 Scoliosis & Spine Unit of "KAT" Orthopaedic Hospital, Athens, Greece; 3 The South West London Elective Orthopaedic Centre, Denbies Wing, Epsom General Hospital, Dorking Road, Epsom, KT18 7EG, United Kingdom

## Abstract

**Background:**

The dynamic derotation brace (DDB) was designed in Greece in 1982, as a modification of the Boston brace. It is a custom-made, underarm spinal orthosis featuring aluminium blades set to produce derotating and anti-rotating effects on the thorax and trunk of patients with scoliosis. It is indicated for the non-operative correction of most curves, barring the very high thoracic ones, (when the apex vertebra is T5 or above). The purpose of this article is to familiarize physicians with the DDB, analyze the rationale behind its design, and present the published results of its application.

**Description & Principles:**

The key feature of the DDB is the addition of the aluminium-made derotating blades posteriorly. These function as a force couple, which is added to the side forces exerted by the brace itself. Corrective forces are also directed through pads. One or more of previously proposed pathomechanical models of scoliosis may underline the corrective function of the DDB: it may act directly on the apical intervertebral disc, effecting correction through the Heuter-Volkman principle; the blades may produce an anti-rotatory element against the deforming "spiral composite muscle trunk rotator"; or it may alter the neuro-motor response by constantly providing new somatosensory input to the patient.

**Results:**

Based on measurements of the Cobb and Perdriolle angles, up to 82% of patients remained stable or improved with the use of the DDB. Results have varied, though, depending on the type/location of the deformity. The overall results showed that 35% of the curves improved, 46% remained stable and 18% became worse, as assessed by measuring the Cobb angle. The DDB has also been shown to improve cosmesis (except for right thoracic curves) and leave several aspects of patient quality of life unaffected during use.

**Conclusion:**

Conservative treatment of idiopathic scoliosis using the DDB has shown favorable results. Thoracic curves appear more resistant to both angular and rotatory correction. The published outcome data on the DDB support our belief that the incorporation of aluminium blades to other orthoses would likely improve their efficacy.

## Background

Viewed in three dimensions, scoliosis is characterized by a constellation of deformities. Ideally, conservative management of scoliosis should aim at correcting simultaneously the lateral deviation of the spine in the frontal plane, the rotational and the rib cage deformity in the transverse plane and restore the sagittal plane.

Brace treatment should be instituted by means of appropriately manufactured orthoses, capable of achieving a satisfactory initial correction. In addition, the corrective forces must be sustained throughout the entire treatment period.

Dynamic Derotation Braces (DDBs) are custom-made, underarm spinal orthoses equipped with specially designed derotating blades that are set to produce a derotating or an anti-rotating effect on the thorax and the trunk of scoliotic patients. *Derotation *is defined as the correction of the rotational deformity (e.g. reduction of Perdriolle angle value). *Anti-rotation *is defined as the prevention of rotational deformity progression. When there is anti-rotation in a progressive curve, the Perdriolle angle remains unchanged during treatment. In other words, progression of vertebral rotation or rib cage rotational deformity in the transverse plane is prevented.

The purpose of this report is to present an overview of the DDB, including the historical background, the biomechanical principles of its function, technical aspects pertinent to its prescription, construction, and the so far published results on its use. The general theoretical principles of conservative treatment with braces will also be presented.

### History

The DDB was designed and used by surgeons of the Scoliosis & Spine Unit of "KAT" Orthopaedic Hospital in Athens and the Certified Orthotist and Prosthetist (CPO), Mr. Nikolaos Vastatzidis of Athens. Based on their extensive experience with different types of braces, this group designed a modification of the classic modular Boston Brace, in order to address the rotational element of scoliosis. The first type of brace they developed was called Boston LP (Limited Pressure). Following its short-lived use, this was replaced by the Dynamic Derotation Brace. The new design, introduced in 1982, was based again on the basic BOSTON BRACE, with the addition of a system of light and slightly flexible blades made of aluminium. The construction of the new brace was based on the traditional plaster mould of the trunk of the patient, onto which the heat-treated PVC sheet was applied. The blades were added last and were placed at the highest point of the hump.

Initial results were very encouraging and, with increasing experience of its application, the brace became more elaborate. Radiological and clinical results showed the new brace to be an effective solution to the conservative treatment of scoliosis and provided evidence of true deformity correction, as opposed to mere curve maintenance. Although the use of this particular type of modification of the Boston Brace started to spread because of its encouraging results, formal presentations on its efficacy were delayed.

For historical and ethical purposes, the then "KAT" Hospital team consisted of Dr. P. Smyrnis (former Head of the Unit), Dr. D. Antoniou (Head of the Unit), Dr. J. Valavanis and Dr. C. Zachariou (both spine surgeons and members of staff).

The first official announcement on the use of the Dynamic Derotating Brace (DDB) was made in 1986 [1]. This brace is now considered the gold standard for the conservative management of idiopathic scoliosis in Greece [2-5].

### The DDB modules - principles of construction used by CPOs

The DDB module is a type of Thoracic Lumbar Sacral Orthosis (TLSO). Its main characteristic is that it is supplied with metallic blade/s on its posterior surface which act as de-rotation or anti-rotatory device/s, as defined above.

**Figure 1:**
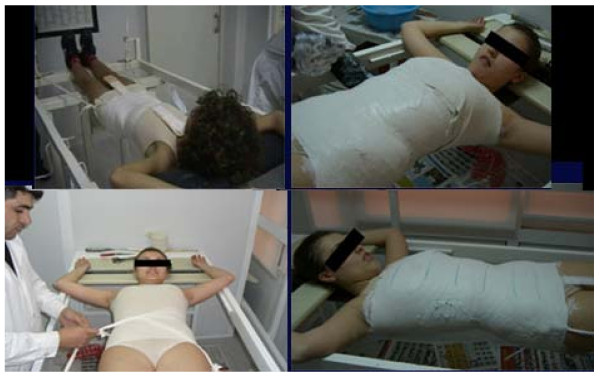
**the DDB production can be based on a cast mould**.

**Figure 2:**
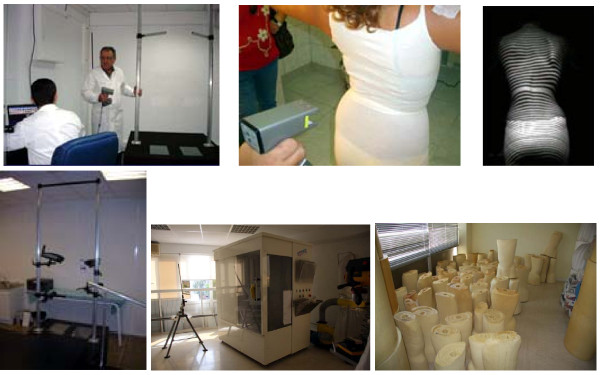
**the DDB production today is based on a pre-trimmed positive plastic template produced after laser scanning of the patient, or raster stereography (CAD-CAM technology)**.

Today, production of the DDB is based on a traditional cast mould, figure 1, or on a pre-trimmed positive plastic trunk template produced after laser scanning of the patient, (CAD/CAM technology) [6-8], figure 2. A blueprint is thus designed, which is a systematic way of analyzing the curve and applying the appropriate force vectors [9].

**Figure 3:**
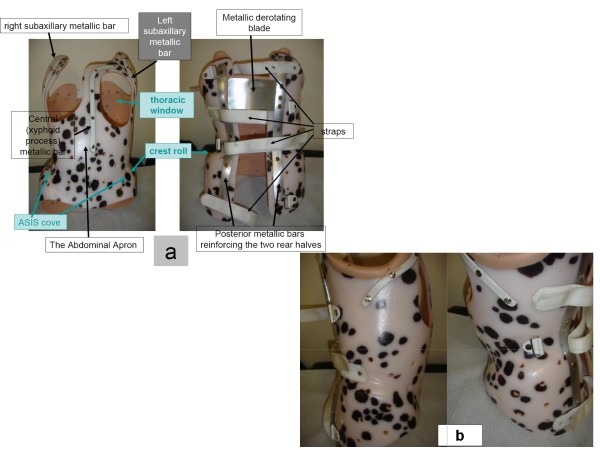
**The DDB extends from underneath the axillae to the pelvis, b: lateral view of a DDB**.

The DDB is a custom-made, underarm hard orthosis, extending from underneath the axilla to the pelvis figures 3a, 3b. The core of the module is made of one 3 mm-thick piece of polyvinylchloride (PVC), which may have an inside lining of plastazote. It opens at the back and is fastened with three or, more commonly, four straps. The design is tailored to the body habitus of the patient. The waist section should be designed to provide maximal patient comfort.

***The axillary extension ***lies just below the shoulder. It should be applied to the lateral surface of the upper part of the sides of the thorax, so as to maintain a direct inward force on this. In essence, it acts by shifting the upper part of the thoracic curve. It comprises the highest lateral component of the DDB module, figure 3a.

***The abdominal apron ***is the front part of the module and extends in a way that includes the abdominal area and covers the edges of the sides and the xyphoid process, taking particular care not to impact on the sides. This part is flat on its front surface, allowing rectification of the body in the module, figure 3a.

Several parts of the brace are reinforced with pre-contoured, aluminium metallic bars (of approximately 1.6 cm in breadth and 0.35 cm in thickness) or bars of similar dimensions made of plastic similar to the plastic sheet of the brace proper. For a brace for a right thoracic curve, the ***right subaxillary metallic bar ***ascends up to the lateral third right subclavicular region, while the ***central metallic bar ***starts from the xiphoid process anteriorly and is turned laterally to the left, merging with the ***left subaxillary metallic bar ***placed opposite to the hump, figure 3a.

The two rear side halves ascend towards the upper border of the scapula, and are spaced approximately 5 cm apart when the brace is on. Both of these posterior halves are reinforced with ***posterior metallic bars ***(approximately 3 cm in breadth and 2.2 mm thick) along their free edges, starting from the scapula down to the pelvis, figure 3a. The pads inside the brace are also lined with plastazote.

The fundamental characteristic of this brace are the ***metallic derotating blades***, figure 3a. These are rectangular, 2.0 - 2.5 mm thick blades, made of aluminium, (semi-rigid aluminium alloy). One side of the blade is fixed along the posterior metallic bar, while the free end is pre-curved to an obtuse angle, opening outside the structure. The amount of this opening is directly proportional to the magnitude of hump. Upon application, the free end of the blade is inserted under the opposite posterior half of the brace. The position of the blades is determined by the level of the hump: they are attached to the rear side of the brace, at the area corresponding to the most prominent area of the thorax or trunk of the patient (thoracic or loin hump; that is, the blade is positioned by the orthotist on the apex of the hump as it is detected clinically).

The blade covers most of the hump prominence. In longer curves, more than one blade may be required. The usual width of a blade is 8-10 cm and their length depends on the patient's somatotype. Hitherto there is no study to document the angle of the blade in relation to the scoliometer measurement. However, the angulation of the blade is done empirically by the very experienced CPOs.

The application of the brace is completed with fastening of four posterior straps, figure 3a.

Once the brace is finally fitted on the patient, the positioning of the blades can be checked on standing radiographs, especially in relation to their correspondence with the apex of the curve. If necessary, the blades can be easily repositioned.

The derotating function of the blades is accomplished through the continuous application of corrective forces at the pressure areas. At the same time, the two posterior halves of the brace move in opposite directions. The force couple thus created is added to the side forces exerted by the brace itself. The direction of action of the blades may be modified, if needed, by altering the (open outside) obtuse angle of the blades [4,5].

The ***trimline ***is usually at the level of the clavicle superiorly. It must cover the anterior superior iliac spine inferiorly, to maximize the lever arm controlling correction in the sagittal plane.

The ***trochanteric extension***, for lumbar modules in particular, is designed so as to extend over the greater trochanter on the side of the convexity of the curve. This increases leverage and facilitates overall balance of the trunk, restoring the alignment of the patient back to neutral, [9].

The ***thoracic extension ***is designed so as to exert an upward and medially directed force at the apex and below; its superior edge should be in line with the contour of the apical rib. Its height is determined by the individual patient's characteristics [9].

The antero-lateral ***thoracic window ***is meant to relieve the patient's torso. It is located directly opposite to the thoracic hump, extending well above the ***crest roll***, which is placed inferiorly, figure 3a, [9].

The ***pads ***are used to direct corrective forces within the DDB system. Their main role is to exert high forces on scoliotic curves. Opposite each force lies an area of relief. They are placed onto the inner surface of the module and apply high pressures at their points of contact with the body [9].

### Curve classification used for prescription purposes

**Figure 4:**
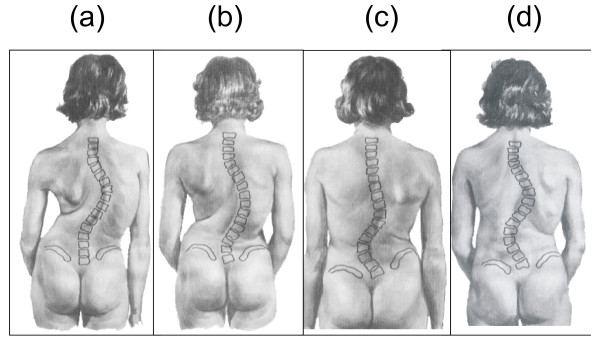
**DDB designs are based on the commonly used classification, which distinguishes scoliotic curves into thoracic (a), thoracolumbar (b), lumbar (c), and double major (d)**.

DDB designs are based on the commonly used classification, which distinguishes scoliotic curves into thoracic, thoracolumbar, lumbar, and double major figure 4.

### Indications - Contraindications

The DDB is indicated for the conservative treatment of the above described curves. Very high thoracic curves (apex at T5 or higher), constitute a contraindication for the application of a DDB.

### Types of DDB designs

There are three main types of DDB designs.

**Figure 5:**
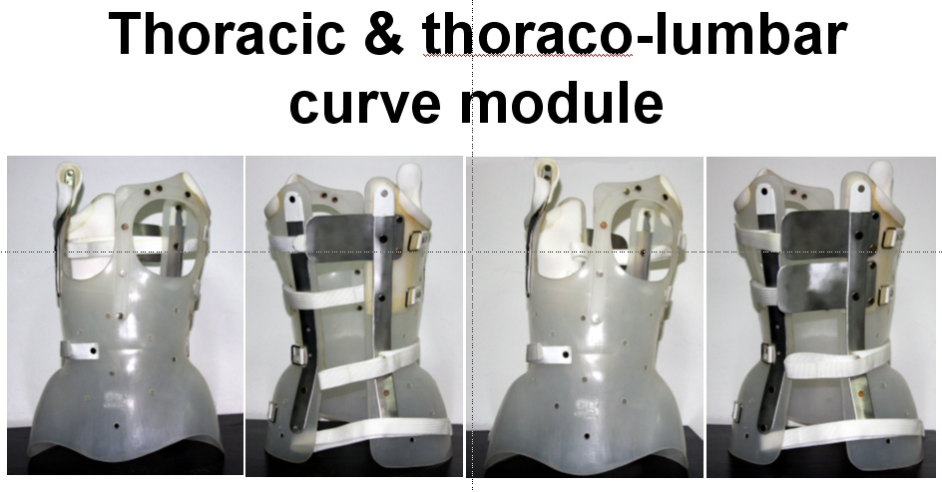
**The thoracic/thoracolumbar curve pattern module encompasses one or two de-rotatory blades, located opposite to the hump**.

The **thoracic/thoracolumbar module**, whose main indications are thoracic or thoracolumbar curves. It encompasses one or two de-rotatory blades, attached opposite to the thoracic or thoracolumbar hump, figure 5.

**Figure 6:**
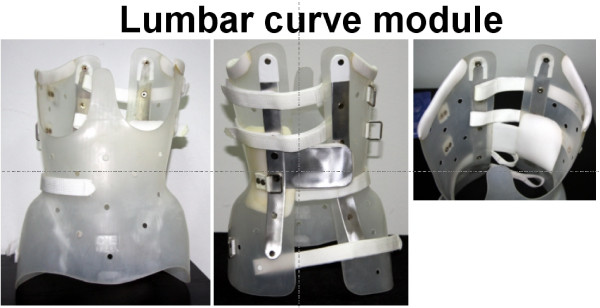
**The lumbar curve pattern module**.

The **lumbar module**, used in lumbar curves, is constructed with one de-rotatory blade, located opposite to the lumbar loin hump, figure 6.

**Figure 7:**
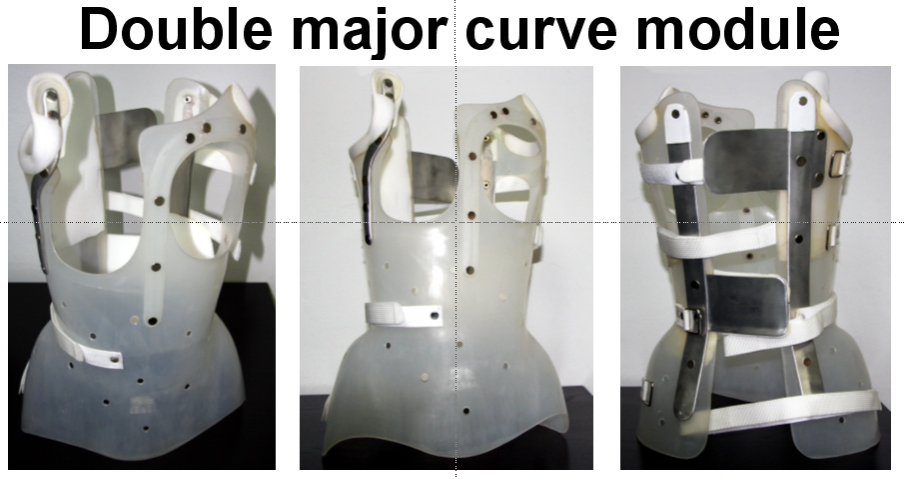
**The double major curve pattern module**.

The **double curve module**, figure 7, used in patients with double major curves, is supplied with two de-rotatory blades, placed over the thoracic hump and lumbar loin hump each. Each blade acts on the contralateral posterior half of the brace.

The detailed construction of each of the described modules is depicted clearly in figures 5, 6 and 7. A major difference of the lumbar curve pattern module with the thoracic/thoracolumbar curve pattern and double major curve pattern modules is the longer trochanteric and the reduced thoracic extension of the former, as seen in figure 6, compared with the later two modules (see figures 5 and 7). The positioning of the de-rotation blade also differs according to the curve pattern module as described. As noted previously, the pads are always placed against the apex of the hump.

### Principles of correction of scoliotic curves with the DDB - Biomechanics

The theoretical principles of DDB function could be associated with one or more of the principles, phenomena and actions described below.

#### a. The Hueter-Volkmann principle, the diurnal variation and the role of the intervertebral discs

The rationale for conservative management of scoliosis during skeletal growth assumes a biomechanical mode of deformity progression, based on the Hueter-Volkmann principle [10], whereby extra axial compression decelerates growth and reduced axial compression accelerates it [11]. In treating scoliosis conservatively, bracing does nothing more than exploiting this principle, by applying appropriately directed forces all through the skin, soft tissues and ribs to the vertebral end-plates.

It has been reported that in mild scoliotic curves, when the deformity is first developing, the intervertebral disc (IVD) is wedged, but the vertebral body is not. Because of the increased plasticity of the IVD, the spine is deformed first at the level of the IVD, in the direction of either torsion or wedging, as an expression of other initiating factors that may result in idiopathic scoliosis (IS) [12,13]. The IVD contains the aggrecans of glycosaminoglycans (GAGs) which imbibe water through the so called Gibbs-Donnan mechanism. The highest concentration of aggrecans is found in the nucleus pulposus (NP), where they are entrapped in a type II collagen network [14]. There is an increased collagen content in the NP of patients with adolescent IS, which is maximal at the apex of the curvature. Furthermore, in the scoliotic spine, the NP in the IVD is displaced towards the convex side of the wedged interspaces [15]. Differences also exist in the collagen distribution between the concave and convex sides of the scoliotic annulus fibrosus in adolescent IS, with depleted levels in the former compared to the latter [16].

By composing all the above findings, it has been suggested [12,13] that the content of imbibed water mainly in the apical IVD, but also in the adjacent discs above and below it, must be higher in the convex that the concave side. This asymmetrical pattern of the water distribution in the scoliotic IVD, in association with the diurnal variation in the water content of lumbar IVD [17], imposes asymmetrical, convex-wise, concentrated cyclical loads to the IVD and the adjacent immature vertebrae of the child during the 24-hour period. The convex side of the wedged IVD sustains greater amount of expansion than the concave side, resulting in asymmetrical growth of adjacent vertebrae (Hueter - Volkmann law). The strong correlation between lumbar Lower InterVertebral Disc Wedging (LIVDW) and thoracic Cobb Angle (CA) reported by Grivas et al in 2006 [12,13] implicates the lumbar spine and particularly the lumbar LIVDW in the progression of a scoliotic curve, as the height of the lumbar IVDs is significantly increased. These correlations [12,13] imply that apical intervertebral disc wedging through this proposed mechanism may be an important contributory factor in the progression of IS curves. This emphasizes the role of the apical intervertebral disc in the pathogenesis of IS [18].

It could thus be postulated that conservative treatment (full-time braces and/or exercises) corrects the deformity of the skeletally immature spine by reversing wedging of the IVD. Modulation of the IVD through the application of corrective forces may restore a close-to-normal force application on the vertebral end-plates and consequently prevent curve progression through the Hueter-Volkmann principle. Under the influence of bracing, the forces are distributed evenly on the end-plate, increasing the proliferation rate of chondrocytes at the corrected pressure side (i.e. the concave). The application of appropriately directed forces, ideally opposite to the apex of the deformity, likely leads to optimal correction. This could reverse the wedging of the elastic IVD in the skeletally immature scoliotic spine. Reversal of IVD wedging is thus amended into a "corrective", rather than "progressive", factor of the deformity. Through the proposed model, treatment of progressive IS with braces, could be effective.

Full-time treatment using a DDB module corrects or even overcorrects the mild or moderate scoliotic curve by acting on the apical and adjacent wedged IVDs, thereby reducing the previously described asymmetrically imbibed water (higher content in the convex than the concave side). Hence, the diurnal variation in the water content of IVD occurs under more favourable conditions. With the action of the DDB, the convex side sustains no greater amount of expansion than the concave side, thus terminating the asymmetrical application of Hueter - Volkmann law and reversing the progression of IS curves. The resultant restoration of a close-to-normal biomechanical environment leads to normal growth of the apical and adjacent immature vertebrae.

#### b. The *flag-pole dinner plate *and the *spiral composite muscle trunk rotator *concepts and the role of the derotating blades

In a previously postulated theory, it was reported that scoliosis resulted from a breakdown of rotational control in the spine caused by the interaction of two mechanisms: a pelvic rotation - inducing system involving gait, femoral anteversion and the pelvis, with rotation transmitted to the lumbar and thoracic spine; and a spinal rotation - defending system involving the intervertebral discs, ligaments, spinal shape in the sagittal plane, the ribs and neuro-muscular mechanisms (the *flag-pole dinner plate *concept). The essential feature of this theory is the failure to control cyclical rotations in the spine during gait [19,20].

This theory integrates the concept of a *spiral composite muscle trunk rotator*. Wemyss-Holden et al. [21] proposed the theoretical concept that rotation of the human trunk during gait is influenced by a spiral composite muscle trunk rotator - comprising the levator scapulae and rhomboids, serratus anterior, and external oblique muscles on each side and the contralateral internal oblique muscle. This concept was originally introduced by Benninghoff [22]. Wemyss-Holden et al. [23] subsequently developed a mechanical model of the composite muscle trunk rotator for scoliosis. It was shown, first, that asymmetry of one or more of the components of the model created an axial deformity; and, secondly, that the site and type of the induced lateral 'spinal' curve was related to the 'muscle' which was shortened [23], and that this would be on the concavity of the thoracic component of a double curve [24]. These findings are consistent with the view that neuromuscular factors are involved in the aetiology of IS [19,20]. In double scoliotic curves or curves with compensatory component (e.g. main thoracic with compensatory lumbar curve), a deforming rotatory force, generated by the asymmetrical action of a component of the above-described spiral composite muscle trunk rotator, is probably present. It is highly likely that the blades of DDB create an anti-rotatory force and block this deforming rotator action.

#### c. Neuro-motor reorganization

The correction achieved by the DDB could also be attributed to an alternative hypothesis, which suggests that the constraint of bracing leads to changes in external and proprioceptive inputs and locomotion, resulting in neuro-motor reorganization [25-28]. According to this hypothesis, braces are considered the driving forces of movement while they increase external and internal bodily sensory input. This permanently changes motor behaviours, even when the brace is removed, and can also have a long-term effect on bone formation. This hypothesis lacks firm supporting evidence, however, and further investigations into its validity are needed [29].

#### d. Mechanical principles of curve correction using a DDB: biomechanics of the derotating blade

The function of the DDB, similar to that of other hard braces, follows concepts of passive and active deformity correction. The brace provides mechanical support to the body (passive correction), and the patient pulls his/her body away from pressure sites (active correction).

The DDB corrects the scoliotic curve through the application of side forces which are transmitted to the spinal column mainly through the costal arches.

**Figure 8:**
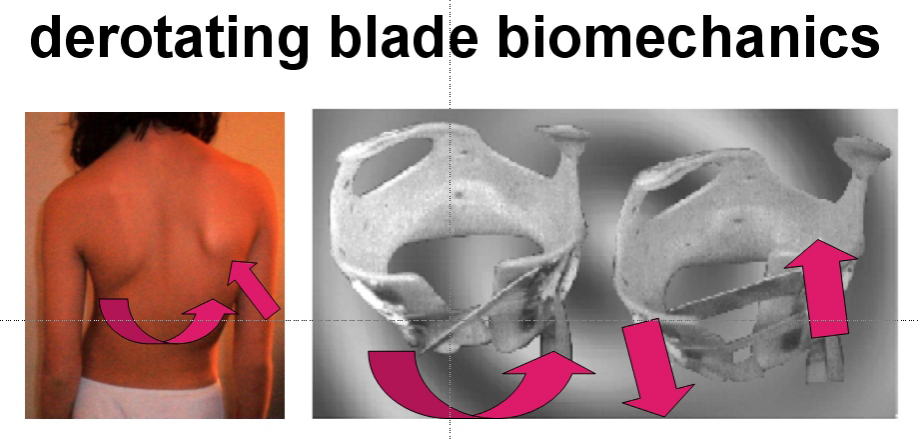
**When the brace is applied, the derotating blades exert anteriorly-directed forces at their fixation points and posteriorly-directed forces under the opposite half of the brace, thus forcing the two halves of the brace to move horizontally in opposite directions to each other**. Therefore, correction in the transverse plane is achieved

When the brace is applied, the derotating blades exert anteriorly-directed forces at their fixation points and posteriorly-directed forces under the opposite half of the brace, thus forcing the two halves of the brace to move horizontally in opposite directions to each other. Therefore, correction in the transverse plane is achieved, figure 8.

The magnitude of forces applied by the derotating blades is added to the correcting forces already exerted by the brace itself and can be controlled by changing the angle of the blades backwards. With the activation of the derotating blades, after fastening the straps, the direction of the correcting forces of the brace is actually transformed from side-to-side to circumferential, adding a derotating element to the overall correcting force, which is exerted mainly upon the rib hump, [4,5].

Concerning the quantification of forces exerted by the blade, studies using cutting-edge, technology, (pressure sensors), are under way, and the results are expected to be published in due course.

### How to prescribe a DDB

In prescribing a DDB, there are several points the physician should consider.

The type of the module (**thoracic/thoracolumbar, lumbar, or double curve module)**, the site of the inner pressure pad(s), and the exact site and number of derotating blades are all important parameters which are individualized for each patient and must be specified by the prescribing physician.

*Example of prescription: *DDB Orthosis (module type according to the curve pattern type) with aluminium reinforcement and derotation blades (the position and number of blades is also specified according to the curve type pattern).

### How to check the brace: principles of checking by MDs and CPOs

The clinical and radiological follow-up assessment is performed immediately after brace construction and first application of the module to the patient. It is critical for the physician to ascertain that the DDB fits perfectly the patient and has no adverse effects on standing balance, [30]. At times, adjustments are needed to render the brace fully operational. The in-brace radiological investigation follows, whose purpose is not only to verify an optimal fit, but also to document the extent to which the curves respond to treatment (correctability of the curve and immediate in-brace correction).

### Protocols and criteria for DDB bracing

Current indications for brace treatment and management are in accordance with the inclusion and assessment criteria for the conservative treatment of scoliosis, as proposed by the SOSORT and the SRS; moreover, they are in accordance with the SOSORT standards of management [31-34].

A change in the Cobb angle by a minimum of 5°, as compared with the reading on the previous radiographs, is accepted as evidence of improvement or deterioration of a curve. Most symmetrical brace designs have found that 50% of initial in-brace Cobb angle correction is a predictor of successful conservative treatment.

Brace use by the scoliotic patients should be full-time, with only 1-2 hours per day allowed out of it for purposes of self-hygiene.

### Everyday usage

The treatment regimen usually involves bracing for 23 hours daily. It is recommended that the parents observe closely the daily use of the brace by the young patient, with special emphasis on the fastening of the straps. This should be done in a consistent manner, preferably by the parents. Marks or arrows drawn by the CPO upon first use are extremely helpful in achieving consistent tightening of the brace on a daily basis. However, during the warmest seasons of the year, patients are allowed to discontinue use of the brace for 3-4 hours daily, should they wish to swim. At school, patients usually have some sessions of physical education (PE). Patient participation is encouraged. Upon completion of a PE session, the straps may be tightened by classmates or teachers, paying attention to the marks and arrows on the outer surface of the straps to ensure that these are always fastened with the same tension, [35].

### Exercises

The DDB group of patients have no exercises to perform during brace treatment.

## Results

The DDB has been evaluated with use of different outcome measures, including radiographic parameters, such as the Cobb angle [36] and the Perdriolle angle of rotation, [37], cosmesis, evaluated by changes in the Angle Trunk Inclination (ATI), and the quality of life, as assessed with the Brace Questionnaire (BrQ), [38,39].

### Cobb angle and Perdriolle angle of rotation

In 1995, Valavanis et al reported an initial correction rate of 49.54%. At two years' follow-up, the correction rate was 44.1% [4]. In 2003, Grivas et al reported an overall improvement rate of 35.7%, while the deformity had remained stable in another 46.42% of their patients. The scoliotic deformity increased in 17.83% of patients in that report [5].

A more detailed analysis of the 28 scoliotics treated conservatively with the DDB in the series of Grivas et al follows:

Seventeen children had double curves (double curve group), four had thoracic curves (thoracic group), and seven had thoracolumbar curve (thoracolumbar group).

In the double curve group, 17 children (15 girls, 2 boys; mean age, 12.3 years; range, 8 - 17) had right thoracic and left lumbar curves with a mean thoracic Cobb angle of 23.2°(range, 10°- 35°) and a mean lumbar Cobb angle of 21.2°(range, 8°- 30°). Using the Perdriolle method, rotation was measured at a mean value of 6.9°(range, 3°- 25°) for the thoracic curve and 7.8°(range, 4°- 15°) for the lumbar curve. The mean follow-up was 28 months (range, 14 - 84).

In the thoracic group, 4 children (all girls; mean age, 13.8 years; range 12 - 15) had right thoracic curves with a mean thoracic Cobb angle of 25°(range, 22°- 31°) and a mean apical vertebral rotation of 6.8°(range, 3°- 10°). The mean follow-up was 8 months (range, 6°- 18°).

In the thoracolumbar group, 7 children (6 girls, 1 boy; mean age, 13.5 years; range, 12 - 17) had thoracolumbar curves with a mean Cobb angle of 24°(range, 20°- 38°) and a mean apical vertebral rotation of 10°(range, 4°- 30°). The mean follow-up was 9 months (range, 6 - 21), [5].

The results presented pertain to the Cobb angle and Perdriolle readings, as measured on follow-up radiographs obtained without the brace.

At final follow-up of the double curve group, 6 curves improved, 7 curves remained stable and 4 curves increased. In five children, curve improvement involved both thoracic and lumbar Cobb angles, whereas in one child only the thoracic Cobb angle improved. In the four children with increase of their scoliosis, this was noted on both the thoracic and the lumbar curves. Concerning rotation at final follow-up, the mean value for the thoracic curves at the apical vertebra was 6.2°(range, 0°- 22°) for thoracic and 5°(range, 0°- 12°) for lumbar curves.

In the thoracic group, the Cobb angle remained unchanged in two curves and improved in another two. The mean rotation at follow-up was 5.4°(range, 2°- 10°).

In the thoracolumbar group, two curves improved, four remained stable and one increased. At final follow-up, the mean rotation measured 8.6°(range, 4°- 20°).

The overall results, including all three (double, thoracic and thoracolumbar) groups, indicate that 10 curves out of 28 (35%) improved, 13 (46%) remained stable and five (18%) became worse, as assessed by measuring the Cobb angle. One (4.3%) patient underwent surgical correction. Four out of five curves that increased were double (right thoracic and left lumbar).

Rotation (measured with use of a Perdriolle template at the apical vertebrae in patients out of the brace) improved only in the lumbar component of the double curves (p < 0.004) and remained unaffected (statistically non-significant change) in the thoracic component of double and in single curves.

By comparison, the Cobb angle of the same children was measured while in-brace. Nineteen (67.8%) curves improved, seven (25%) remained stable and two curves (7.1%) increased. When children with double curves were wearing the brace, the mean apical vertebral rotation measured 5.8°and 3°for the thoracic and lumbar curves, respectively. An additional de-rotating effect for the lumbar component was thus clearly observed. For further details on this study, the reader is referred to Table one of the publication by Grivas et al (2003) [5].

### Cosmesis

Grivas and Vasiliadis (2008), in their study of the Angle Trunk Inclination (ATI) - hump, reported that the DDB improved the cosmetic appearance in all children with IS but those with right thoracic curves, [38].

### Quality of life

The study of QoL using the Brace Questionnaire (BrQ), [39], revealed that the DDB had a negative impact on school activity & social functioning. However, there were no adverse sequelae on general health perception, physical functioning, emotional functioning, vitality, bodily pain, self-esteem & aesthetics, [40,41].

## Cases presentation

**Figure 9:**
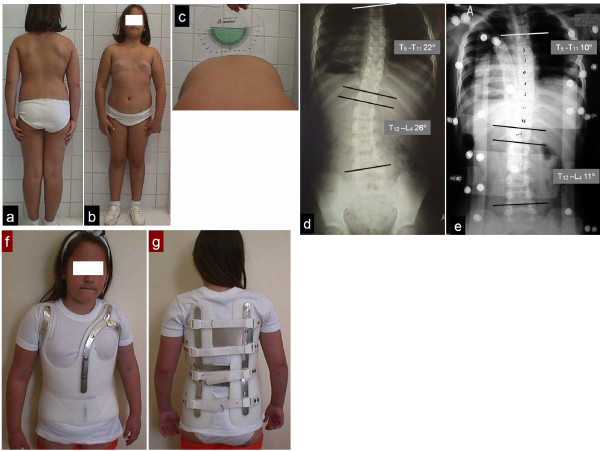
**Figure 9a front and 9b back view of a pre-menarche scoliotic child, 8 years of age with Idiopathic Scoliosis, 9c scoliometer reading 7 degrees, 9d radiographic study: double curve, Risser 0, T5 - T11: 22 degrees Cobb angle, T12 - L4: 26 degrees Cobb angle, 9e immediate post brace application correction, T4 - T11: 10 degrees Cobb angle (54,5% correction), T12 - L4: 11 degrees Cobb angle (57,7% correction), 9f anterior and 9g posterior view of the girl with the DDB on, a double major curve module with two derotation blades, (thoracic and lumbar)**.

**Figure 10:**
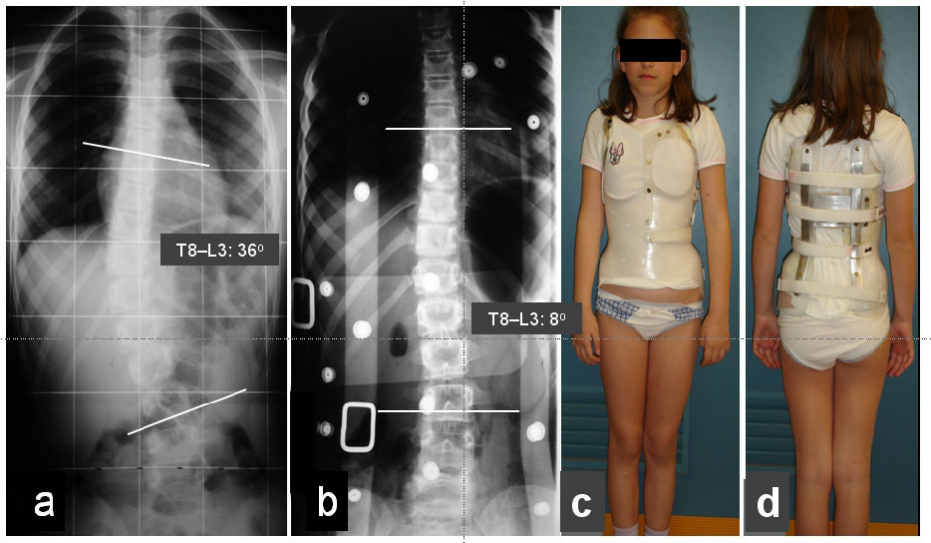
**a thoraco-lumbar scoliotic curve in a 8,5 years old girl, Risser 0, T8-L3: 36 degrees Cobb angle, b, immediate post brace application correction, T8 - L3: 8 degrees Cobb angle (77,8% correction), c, anterior and d, posterior view of the girl with the DDB on, (a thoracic/thoraco-lumbar curve module with one derotation blade)**.

The use of DDB is depicted in two scoliotic girls receiving conservative treatment, figure 9 and figure 10, (see figure legends for details).

## Discussion

The findings of the previously presented study [5] demonstrate that the majority of curves were positively affected by the treatment. This study on the DDB showed that the action of the brace prevailed over the deleterious process of the pathology in these curve patterns. The application of the DDB seems to have a positive effect on the natural history of IS.

Similar beneficial outcomes of the use of the DDB on the natural history of those curves have been reported by Valavanis et al. [4], thus validating the results and confirming the reliability of the brace.

With regards to rotation, as measured with a Perdriolle template at the apical vertebra in children out of the brace, improvement was noted in the lumbar component of double curves (p < 0.004) and rotation remained unaffected (statistically non-significant change) in the thoracic component of double and in single curves. These findings verify the beneficial effect of the derotating blades (anti-rotatory potential, in this case) in halting the progression of vertebral rotation.

Apart from their de-rotating (correction) or anti-rotation (prevention of progression) effect, the blades of the DDB may possess another mode of function. It is likely that, by creating an anti-rotatory force, they block the deforming rotatory action of the spiral composite muscle trunk rotator (mentioned above). The counter-action of the anti-rotatory blades to the rotation-inducing system on the lumbar component of double (right thoracic - left lumbar) curves also indicates that failure to control cyclical rotations may be a major aetiopathogenic factor for those curves.

The results on cosmesis of the DDB demonstrate the inherent insufficiency of underarm TLSOs in properly controlling the thoracic hump. This is a limitation of this brace and points to the need for improvement in that direction. Research on this issue is mandatory.

The results on quality of life, [39] highlight the need for new methods which will diminish the negative impact of bracing (including the DDB) on school activity & social functioning. There is definitely room for future research on this topic. That said, it is very encouraging that the general health perception, physical functioning, emotional functioning, vitality, bodily pain, self-esteem & aesthetics were not affected by the treatment, [40,41].

## Conclusions

In our opinion, the advantages of the DDB far outweigh some limitations associated with its application. We further believe that the DDB is highly recommended for the conservative treatment of idiopathic scoliosis.

This report could serve as a framework for the presentation of other available brace technologies, in order to increase awareness and understanding of bracing for idiopathic scoliosis, as was also highlighted in earlier publications [42].

It is our belief that the principle of derotation may be extrapolated to other brace systems, whose efficacy could potentially be enhanced by the incorporation of one or more derotating blades into them.

## Definitions

*Trimline: *The outline of the brace. Usually high-grade HyperFoam Trimline wraps provide comfort and durability.

*Thoracic extension: *the extension of the brace in the thoracic region

*Trochanteric extension: *the extension of the brace in the trochanteric region

## Consent statement

Written informed consent was obtained from the patients or their parents/legal guardians for publication of this case report and accompanying images. A copy of the written consent is available for review by the Editor-in-Chief of this journal.

## Competing interests

The authors declare that they have no competing interests.

## Authors' contributions

TBG design of the study carried out the literature research and drafted the manuscript AB participated in drafting of the manuscript. IV carried out some of the literature research, NVB helped to draft the manuscript. All authors read and approved the final manuscript.

## References

[B1] Antoniou D Valavanis J Zachariou C Smyrnis P Dynamic Derotation Brace (DDB) A new aspect for the conservative treatment of Idiopathic Scoliosis Presentation in 21st common meeting of SRS and BSS 1986

[B2] Valavanis I Makridis N Papadakis P Tsafantakis E Derotating Thoracic Brace. A new method of conservative treatment of idiopathic scoliosis Presentation in 4th Panhellenic Congress of Greek Orthopaedic Surgeons, 1990, Athens, Greece

[B3] Andoniou D Valavanis J et al The effectiveness of our bracing system in the conservative treatment of idiopathic scoliosis J Bone Joint Surg 1992 74B Suppl, I 86

[B4] Valavanis J Bountis A Zachariou C Kokkonis D Anagnostou D Giahos D Daskalakis E Three-Dimensional Brace Treatment for Idiopathic Scoliosis Three Dimensional Analysis of Spinal Deformities M D'Amico et al 1995 IOS Press 337 340

[B5] Grivas TB Vasiliadis E Chatziargiropoulos T Polyzois VD Gatos K The effect of a modified Boston brace with anti-rotatory blades on the progression of curves in idiopathic scoliosis: aetiologic implications Pediatr Rehabil 2003 6 3-4 237 242 14713591 10.1080/13638490310001636808

[B6] Wong MS Cheng JCY Wong MW So SF "A work study of the CAD/CAM method and conventional manual method in the fabrication of spinal orthoses for patients with adolescent idiopathic scoliosis." Prosthetics and Orthotics International 2005 29 93 104 10.1080/17461550500066782 16180382

[B7] Wong MS Cheng JCY Lo KH "A comparison of treatment effectiveness between the CAD/CAM method and the manual method for managing adolescent idiopathic scoliosis." Prosthetics and Orthotics International 2005 29 105 111 10.1080/17461550500069547 16180383

[B8] Wong MS Lee JTC Luk KDK Chan LCK Effect of different casting methods on adolescent idiopathic scoliosis Prosthetics Orthotics International 2003 27 121 131 10.1080/03093640308726668 14571942

[B9] Wynne JH Grivas TB The Boston Brace System. Philosophy, biomechanics, design and fit The Conservative Scoliosis Treatment. 1st SOSORT Instructional Course Lecture Book. Stud Health Technol Inform 2008 135 370 384 18401105

[B10] Stokes IA Analysis and simulation of progressive adolescent scoliosis by biomechanical growth modulation Eur Spine J 2007 16 10 1621 1628 10.1007/s00586-007-0442-7 17653775 PMC2078290

[B11] Mente PL Stokes IA Spence H Aronsson DD Progression of vertebral wedging in an asymmetrically loaded rat tail model Spine 1997 22 12 1292 1296 10.1097/00007632-199706150-00003 9201830

[B12] Grivas TB Vasiliadis E Malakasis M Mouzakis V Segos D Intervertebral disc biomechanics in the pathogenesis of idiopathic scoliosis6th Biennial Meeting of the International Research Society of Spinal Deformities (IRSSD), The Cultural and Congress Centre of the University of Ghent 'Het Pand', Ghent, Belgium 2006

[B13] Grivas TB Vasiliadis E Malakasis M Mouzakis V Segos D Uyttendaele D, Dangerfield PH Intervertebral disc biomechanics in the pathogenesis of idiopathic scoliosis Stud Health Technol Inform 2006 123 80 83 Research into Spinal Deformities 5 17108407

[B14] Melrose J Gurr KR Cole TC Darvodelsky A Ghosh P Taylor TK The influence of scoliosis and ageing on proteoglycan heterogeneity in the human intervertebral disc J Orthop Res 1991 9 1 68 77 10.1002/jor.1100090110 1984051

[B15] Taylor TKF Melrose J Burwell G, Dangerfield PH, Lowe TG, Margulies YJ The role of the intervertebral disc in adolescent idiopathic scoliosis Etiology of adolescent idiopathic scoliosis: Current trends and relevance to new treatment approaches 2000 Philadelphia: Hanley and Belfus Inc

[B16] Bushell GR Ghosh P Taylor TK Sutherland JM The collagen of the intervertebral disc in adolescent idiopathic scoliosis J Bone Joint Surg Br 1979 61-B 4 501 508 500764 10.1302/0301-620X.61B4.500764

[B17] Dangerfield PH Roberts N D'Amico M, Santambrogio GC, Merolli A Investigation of the diurnal variation in the water content of the intervertebral disc using MRI and its implication for scoliosis Three-dimensional analysis of spinal deformities 1995 Amsterdam: IOS Press 447 451

[B18] Grivas TB Vasiliadis ES Rodopoulos G Bardakos N The role of the intervertebral disc in correction of scoliotic curves. A theoretical model of idiopathic scoliosis pathogenesis Stud Health Technol Inform 2008 140 33 36 18809995

[B19] Burwell RG Dangerfield PH Findlay G, Owen R Pathogenesis and assessment of scoliosis Surgery of the spine, section 5, spinal deformity 1992 Oxford: Blackwell Scientific Publications 365 408

[B20] Burwell RG Cole AA Cook TA Grivas TB Kiel AW Moulton A Thirlwall AS Upadhyay SS Webb JK Wemyss-Holden SA Whitwell DJ Wojcik AS Wythers DJ Pathogenesis of idiopathic scoliosis. The Nottingham Concept Acta Orthop. Belgica 1992 58 suppl. 1 33 58 1456018

[B21] Wemyss-Holden SA Butcher CA Burwell RG Webb JK Moulton A Segmental evaluation of rotational (torsional) deformity in scoliosis: clinicoanatomical observations and surgical significance Clinical Anatomy 1990 4 387

[B22] Benninghoff A Anatomie Band 1, Herausgegeben von J Stoubesand 1985 14 Munich: Urban & Schwarzenberger

[B23] Wemyss-Holden SA Burwell RG Cook TA Binch C Webb JK Moulton A A spiral 'Composite Muscle Trunk Rotator' in man? Relevance to gait, idiopathic and sportsman's scoliosis and stroke Proceedings of British Association of Clinical Anatomists, 20 December, London. Clin. Anat 1990 4 386

[B24] Thirlwall AS Burwell RG Moulton A Webb JK Wemyss-Holden SA The pattern of asymmetry of rib-vertbra angles by curve type in adolescent idiopathic scoliosis Procceedings of British Association of Clinical Anatomists, 18 December, London. Clin. Anat 6 260 199

[B25] Coillard C Leroux MA Badeaux J Rivard CH SPINECOR: a new therapeutic approach for idiopathic scoliosis Stud Health Technol Inform 2002 88 215 217 15456035

[B26] Odermatt D Mathieu PA Beausejour M Labelle H Aubin CE Electromyography of scoliotic patients treated with a brace J Orthop Res 2003 21 5 931 936 10.1016/S0736-0266(03)00038-X 12919883

[B27] Negrini S Marchini G Tomaello L The Sforzesco brace and SPoRT concept (Symmetric, Patient-oriented, Rigid, Three-dimensional) versus the Lyon brace and 3-point systems for bracing idiopathic scoliosis Stud Health Technol Inform 2006 123 245 249 17108434

[B28] Smania N Picelli A Romano M Negrini S Neurophysiological basis of rehabilitation of adolescent idiopathic scoliosis Disabil Rehabil 2008 30 10 763 771 10.1080/17483100801921311 18432434

[B29] Negrini S Grivas TB Introduction to the "Scoliosis" Journal Brace Technology Thematic Series: increasing existing knowledge and promoting future developments Scoliosis 2010 5 2 10.1186/1748-7161-5-2 20205874 PMC2827401

[B30] Sadeghi H Allard P Barbier F Gatto L Chavet P Rivard CH Hinse S Simoneau M Bracing has no effect on standing balance in females with adolescent idiopathic scoliosis Med Sci Monit 2008 14 6 CR293 298 18509271

[B31] Richards BS Bernstein RM D'Amato CR Thompson GH Standardization of criteria for adolescent idiopathic scoliosis brace studies: SRS Committee on Bracing and Nonoperative Management Spine 2005 30 18 2068 75 discussion 2076-7 10.1097/01.brs.0000178819.90239.d0 16166897

[B32] Thompson GH Richards BS Inclusion and assessment criteria for conservative scoliosis treatment Stud Health Technol Inform 2008 135 157 63 18401088

[B33] Weiss HR Negrini S Rigo M Kotwicki T Hawes Mc Grivas TB Maruyama T Landauer F Indications for Conservative Management of Scoliosis (SOSORT Guidelines) Scoliosis 2006 1 5 10.1186/1748-7161-1-5 18401089

[B34] Negrini S Grivas TB Kotwicki T Rigo M Zaina F International Society on Scoliosis Orthopaedic and Rehabilitation Treatment (SOSORT) Guidelines on "Standards of management of idiopathic scoliosis with corrective braces in everyday clinics and in clinical research": SOSORT Consensus 2008 Scoliosis 2009 4 2 10.1186/1748-7161-4-19 19149877 PMC2651850

[B35] Korovessis P Kyrkos C Piperos G Soucacos PN Effects of Thoracolumbosacral Orthosis on Spinal Deformities, Trunk Asymmetry, and Frontal Lower Rib Cage in Adolescent Idiopathic Scoliosis Spine 2000 25 16 2064 2071 10.1097/00007632-200008150-00010 10954637

[B36] Cobb JR Outline for the study of scoliosis AAOS Instructional Course Lecture 1948 5 261 275

[B37] Perdriolle R La scoliose: son étude tridimensionnelle 1979 Maloine, Paris

[B38] Grivas TB Vasiliadis ES Cosmetic outcome after conservative treatment of idiopathic scoliosis with a dynamic derotation brace Stud Health Technol Inform 2008 135 387 392 18401106

[B39] Vasiliadis E Grivas TB Gkoltsiou K Development and preliminary validation of Brace Questionnaire (BrQ): a new instrument for measuring quality of life of brace treated scoliotics Scoliosis 2006 1 7 10.1186/1748-7161-1-7 16759366 PMC1481574

[B40] Vasiliadis E Grivas TB Savvidou O Triantafyllopoulos G The influence of brace on quality of life of adolescents with idiopathic scoliosis Stud Health Technol Inform 2006 123 352 356 17108451

[B41] Vasiliadis E Grivas TB Quality of life after conservative treatment of adolescent idiopathic scoliosis Stud Health Technol Inform 2008 135 409 13 18401108

[B42] Rigo M Negrini S Weiss HR Grivas TB Maruyama T Kotwicki T SOSORT 'SOSORT consensus paper on brace action: TLSO biomechanics of correction (investigating the rationale for force vector selection)' Scoliosis 2006 1 11 10.1186/1748-7161-1-11 16857045 PMC1553475

